# Mortality risk stratification based on comorbidity status among cervical cancer patients in Lagos, Nigeria

**DOI:** 10.1093/inthealth/ihaf008

**Published:** 2025-02-08

**Authors:** Idris O Ola, Adeyemi A Okunowo, Muhammad Y Habeebu

**Affiliations:** Department of Clinical and Community Service, Blue-Pink Center for Women's Health, Lagos 100361, Nigeria; Department of Obstetrics and Gynaecology, College of Medicine, University of Lagos, Lagos 100254, Nigeria; Department of Obstetrics and Gynaecology, Lagos University Teaching Hospital, Lagos 100254, Nigeria; Department of Radiotherapy, College of Medicine, University of Lagos, Lagos 100254, Nigeria; Department of Radiotherapy, Lagos University Teaching Hospital, Lagos 100254, Nigeria; Lead Oncologist, NSIA-LUTH Cancer Centre, Lagos 100254, Nigeria

**Keywords:** cancer epidemiology, cancer mortality, cancer of the uterine cervix, comorbidity, mortality risk stratification, women's health

## Abstract

**Background:**

Comorbidity amplifies mortality risk by approximately sixfold in cancer patients and affects about 26% of cervical cancer (CC) patients in Nigeria. However, its impact on CC outcomes has yet to be fully explored.

**Methods:**

We analysed data from the Lagos University Teaching Hospital and the NSIA-LUTH Cancer Center in Lagos, Nigeria, between January 2015 and December 2021. Based on the hypertension-augmented Charlson comorbidity index (hCCI), the hazard ratios (HRs) associated with CC mortality were estimated using Cox proportional hazards model.

**Results:**

Our results showed a mortality rate of 30.1/100 women-years with a mean age at death of 59.8 years. Women with hCCI 2–6 had a significant increase in mortality risk in unadjusted (HR 1.68 [95% confidence interval {CI} 1.10 to 2.57]) and age-adjusted models (adjusted HR 1.57 [95% CI 1.02 to 2.42]) compared with those with hCCI 0. When CC stage was considered, the mortality risk gradient by hCCI was pronounced for late-metastatic CC with hCCI 2–6 (HR 2.32 [95% CI 1.23 to 4.39], increasing to 4.15 (95% CI 1.69 to 10.18) in the adjusted model compared with hCCI 0.

**Conclusions:**

Cervical cancer mortality risk increases with an increasing comorbidity score. Routine incorporation of comorbidity scoring in the clinical assessment of CC patients as well as the use of multidisciplinary cancer care teams may positively impact their clinical and psychosocial management.

## Introduction

With an annual global incidence exceeding 600 000 new cases and a substantial mortality surpassing 340 000, cervical cancer (CC) persists as a formidable global health challenge.^1^ This burden is especially pronounced in low- and middle-income countries (LMICs),^1^ with mortality reaching as high as 16.5–28.9 per 100 000 women in sub-Sahara Africa compared with 2.1–6.3 per 100 000 women in Europe and 1.4 per 100 000 women in Australia–New Zealand.^2^ Cervical cancer accounts for approximately 15% of all cancer-related deaths among Nigerian women,^3^ with an estimated 5-y prevalence of 22.11 per 100 000 women.^4^

In the Nigerian context, cancer seldom occurs in isolation. Typically, >25% of cancer patients in Nigeria, and approximately 26% specifically among CC patients, present with comorbidities ranging from prevalent conditions like hypertension and diabetes to infectious diseases such as human immunodeficiency virus (HIV)/acquired immunodeficiency syndrome (AIDS). These comorbidities exert a substantial influence on the treatment outcomes and prognosis of affected individuals.^5^ Notably, relative to those without comorbidities, cancer patients with associated comorbidities face up to a sixfold higher risk of mortality.^6^

The utilization of comorbidity scores has been validated as an efficient method for comprehensively investigating the impact of comorbid conditions on disease outcomes, potentially obviating the necessity for individual comorbidity variables.^7^ In quantifying the influence of comorbidity on cancer outcomes, the Charlson Comorbidity Index (CCI) or its derivatives emerge as commonly employed metrics consistently deemed most suitable, as exemplified in previous studies on cancer.^8^ Furthermore, while a higher comorbidity score correlates with increased cancer mortality risk,^8^ hypertension and hypertension-augmented CCI (hCCI) have strongly influenced survival outcomes, particularly among individuals with advanced-stage disease.^9^ It is noteworthy that hypertension stands as the predominant comorbidity among Nigerian cancer patients.^5,10^

An exhaustive online literature search revealed a solitary report documenting comorbidity prevalence among CC patients as part of a broader investigation into comorbidities across various cancer types in Nigeria.^5^ To our knowledge, no current study in Nigeria specifically quantifies the impact of comorbidities on CC mortality risk, despite its high prevalence and the recognized need for a multidisciplinary treatment approach.^5,10^ Recognizing the existing lack of consensus regarding the interpretation and management of comorbidities coexisting with cancer, Sarfarti et al.^11^ advocated for enhanced evidence-based development of clinical tools to aid decision-making and improved integration and coordination of care for patients with comorbidities.^11^ The lack of in-depth analysis regarding the predictive value of comorbidities could potentially deprive clinicians of the opportunity to precisely assess the impact that varying comorbidity burdens may exert on CC patients.

This study aimed to estimate the impact of comorbidities on the mortality risk among CC patients and explore the potential for integrating comorbidity assessments into multidisciplinary management strategies to improve patient outcomes in Nigeria. By determining the prevalence of comorbid conditions and estimating the effect size of comorbidity on CC mortality risk, our findings could assist clinicians in developing individualized risk stratification and targeted treatment and supportive services to improve survival in CC patients in Nigeria.

## Methods

### Study design and study site

A retrospective cohort study was conducted using data from the medical records of CC patients at the gynaecological oncology unit of the Department of Obstetrics and Gynaecology, Lagos University Teaching Hospital (LUTH) and the Nigerian Sovereign Investment Authority (NSIA)-LUTH Cancer Center (NLCC), Lagos, Nigeria.

The LUTH is a tertiary referral centre with advanced capacity for specialized cancer management services. It receives referrals from all six geopolitical regions in Nigeria. Since 29 May 2019, the NLCC, an advanced cancer treatment centre in the LUTH, has been operating through a public–private partnership between the LUTH and the NSIA Healthcare Development and Investment Company.^12^

### Data source and collection

With support from trained staff and research assistants, a manual search of CC cases from the medical records departments of both the LUTH and NLCC was carried out in October 2022 to retrieve all CC cases seen between 1 January 2015 and 31 December 2021. Relevant information for the study was extracted from the medical records of eligible study participants using structured and pretested forms.

All patients with histologically confirmed CC who received care at the gynaecological oncology unit of the Department of Obstetrics and Gynaecology and the NLCC during the study period were included in the study. Records that were unavailable in the medical records department at the time of data collection (lost, destroyed or transferred to other clinics or wards at the time of our search) were excluded from the study.

The biodata section of the records and clinician documentation provided relevant data about the patients’ sociodemographic characteristics. Information on anaemia and HIV/AIDS status was obtained from the earliest baseline laboratory investigation reports and clinical documentation. The packed cell volume (PCV) was regarded as normal or no anaemia if PCV was ≥36% at presentation. Anaemia was classified using PCV at presentation of ≤35.9% according to the standardized World Health Organization scale for non-pregnant women ≥15 y of age.^13^

Treatment data and information on comorbidity were extracted from the review of the physician's documentation in the patient's case record with emphasis on the medical history, treatment administered and report of medications administered as documented in the nurses’ report notes.

The histological characteristics of CC were obtained from the histology report of the pathologist and were broadly categorized into squamous cell carcinoma, adenocarcinoma and other types based on the sample sizes obtained for each histotype. Staging was done using findings from clinical examination, radiological and pathology reports. CC staging was categorized into early (FIGO I–IIA) and late disease (FIGO IIB–IVB) following the 2018 recommendations of the International Federation of Gynaecology and Obstetrics (FIGO).^14^ Based on this FIGO template and previous research,^15^ we further subclassified late diseases into late non-metastatic (FIGO IIB–IIIC) and late metastatic CC (FIGO IVA and IVB) to better quantify the effect sizes on CC death.^15^

### Measures

#### Exposure and outcome variables

The primary exposure variable was the histological diagnosis of CC, while the comorbidity status of the patient served as a secondary exposure variable. The primary endpoint of interest was the occurrence of mortality during the follow-up period. Occurrence of mortality was assessed by reviewing the death certificate register, death summary form, physician's documentation in the case record and documentation in the nursing process report. In extreme situations where the patient outcome could not be ascertained from any clinical records, phone calls were made to the patient's next of kin to inquire about the patient's clinical state and survival. This method relies on the proven accuracy and reliability of the use of verbal autopsies in assessing deaths in low-income settings.^16^

Follow-up began with a histological diagnosis of CC and ended with the death of the patient, loss to clinic follow-up, referral for continuation of treatment outside the LUTH or NLCC or the common closing date of 31 December 2021, whichever came first. Study participants without CC mortality during the follow-up period were right-censored.

#### Covariates

The sociodemographic, clinical and histological variables were included as covariates in the study. However, only anaemia, age, family history of CC in first-degree relatives, CC stage and types of comorbidity were adjusted for in the final multivariable model, because the other variables did not improve the model fit.

#### Comorbidity score and analytical approach

Data on comorbidity were based on the Deyo modification of the CCI.^17^ For our analysis, we adopted the hCCI proposed by Jung et al.^9^ by including hypertension as a comorbid condition in the CCI and adding an extra point to the standard CCI when a patient has hypertension. In this case, age was separately adjusted for, in addition to other covariates. We conducted further analysis based on an age- and hypertension-augmented CCI (a-hCCI), where 1 point is added to the hCCI for each decade of life after 40 y.^5^

Given the strong influence of cancer stage at diagnosis on treatment plans and survival outcomes, stage-specific analyses have been suggested to enhance the understanding of the role of comorbidity in cancer outcomes.^6^ Therefore, mortality risk by hCCI and a-hCCI was also analysed by the CC stage at presentation.

### Data analysis

The sociodemographic and clinical characteristics of the study population were statistically described. Cox proportional hazards regression models were used for inferential statistics, with hazard ratios (HRs) and their 95% confidence intervals (CIs) as measures of association between CC mortality and the comorbidity status of the patients.

The logrank tests of equality were used for initial univariate analyses, and covariates with p-values <0.25 were subsequently used in the multivariable model.^18^ The proportionality assumption was checked using Schoenfeld and scaled Schoenfeld residuals with a satisfactory global test result of 0.9879. The Akaike information criterion (AIC) was used to select the number of variables adjusted for in the final model and the goodness-of-fit of the final model was checked using the Cox–Snell residuals in a Nelson–Aalen cumulative hazard function.

**Figure 1.**
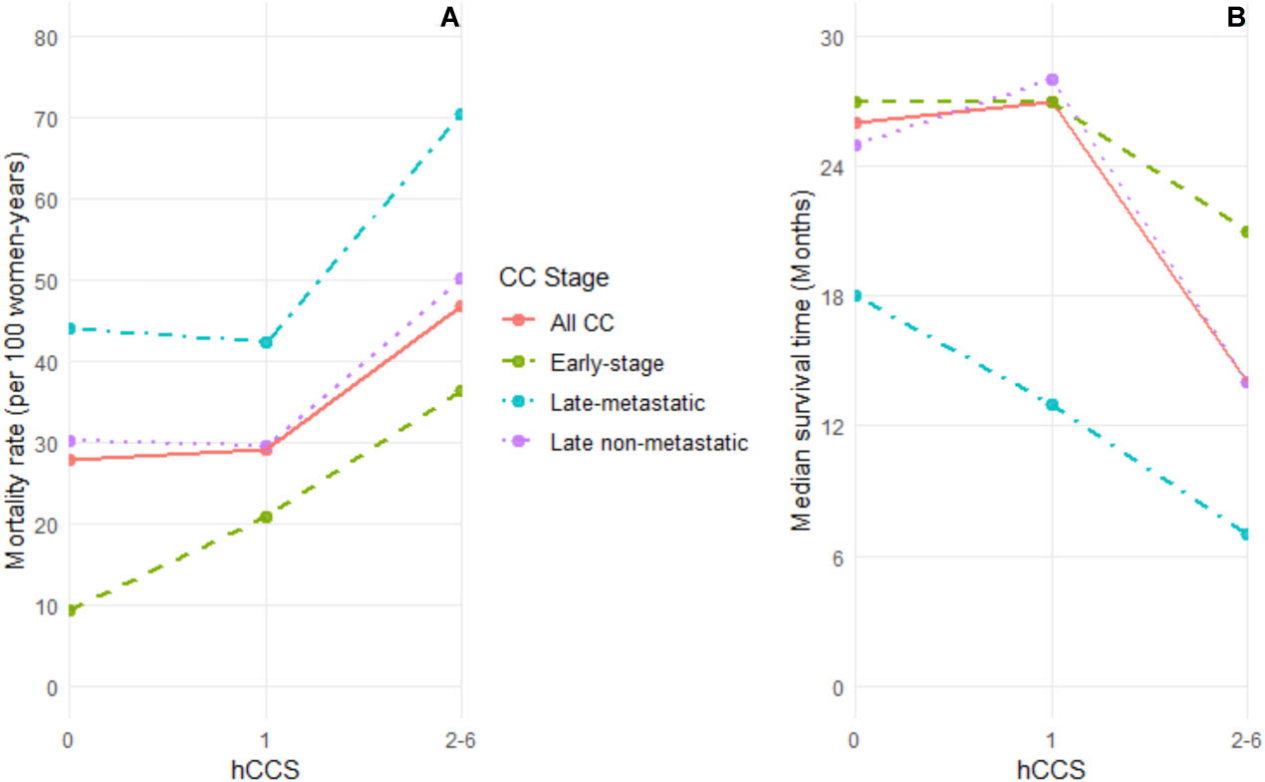
Showing the **(A)** mortality rate and **(B)** mean survival time stratified by hCCI for all CC cases and different CC stages.

All analyses were conducted with Stata SE version 17.0 (StataCorp, College Station, TX, USA) and all tests were two-sided, with a p-value <0.05 taken as statistically significant. The ggplot2 function in RStudio version 4.3.2 (RStudio, Boston, MA, USA) was used to produce Figure 1. The listwise deletion approach was applied in the analysis of each variable of interest with missing information.

## Results

Overall, 350 women met the study criteria and had most of the relevant data at the time of the study. The total estimated time at risk was 644.80 y and the overall median survival time was 25.7 months (interquartile range 12–57.6). The mean age at diagnosis was 55.4 y (standard deviation [SD] 12.5).

Table 1 provides an overview of the sociodemographic and clinical characteristics of the study population. Most cancer diagnoses were recorded among women 51–60 y of age (n=100 [29.5%]). Nearly two-thirds (n=196 [61.1%]) were married in a monogamous setting, more than half (n=184 [57.7%]) had at least a high school education and a majority (79.4%) were employed and of Yoruba ethnicity (40%). Most patients neither smoked cigarettes (95.2%) nor drank alcohol (82.2%). Of the study population, 43.1% (n=147) had at least one comorbid condition and 2% (n=5) had a family history of CC in a first-degree relative.

**Table 1.** Description of the relevant sociodemographic and clinical characteristics of the study cohort including mortality within each group and results of univariate analyses.

Characteristics	Frequency, n (%)	Total CC mortality, n (%_row_)^a^	Logrank test
Age (years) (mean 55.4 y [SD 12.5])	N=339	N=198	
≤40	51 (15.0)	31 (60.8)	0.1045^b^
41–50	70 (20.7)	39 (56.5)	
51–60	100 (29.5)	56 (58.3)	
61–70	82 (24.2)	48 (60.8)	
≥71	36 (10.6)	24 (66.7)	
Marital status	N=321	N=189	
Never married	7 (2.2)	5 (71.4)	0.1328^b^
Widowed/divorced/separated	44 (13.7)	29 (65.9)	
Married (monogamous)	196 (61.1)	103 (54.2)	
Married (polygamous)	74 (23.1)	52 (71.2)	
Education	N=319	N=184	
Less than high school	135 (42.3)	85 (65.4)	0.0662^b^
High school or higher	184 (57.7)	99 (54.7)	
Occupation	N=344	N=200	
Unemployed/unclassified	69 (20.1)	41 (59.4)	0.5087
Employed	275 (79.9)	159 (57.8)	
Ethnicity	N=350	N=205	
Yoruba	140 (40.0)	90 (65.7)	0.0017^b^
Igbo	86 (24.6)	47 (56.0)	
Other	124 (35.4)	68 (57.1)	
Location	N=265	N=164	
Rural	9 (3.4)	3 (37.5)	0.4930
Suburban	159 (60)	102 (65.8)	
Urban	97 (36.6)	59 (63.4)	
Family history	N=250	N=155	
No family history of CC	245 (98.0)	151 (63.2)	0.0146^b^
Family history in first-degree relatives	5 (2.0)	4 (80.0)	
Alcohol use	N=331	N=201	
Yes	59 (17.8)	41 (70.7)	0.1686^b^
No	272 (82.2)	160 (60.2)	
Cigarette smoking	N=331	N=201	
Yes	16 (4.8)	188 (61.0)	0.0407^b^
No	315 (95.2)	13 (81.3)	
Anaemia	N=295	N=185	
No anaemia	50 (17.0)	20 (41.7)	0.0339^b^
Anaemia	245 (83.1)	165 (68.5)	
CC Stage	N=289	N=177	
Early stage	39 (13.5)	15 (38.5)	<0.0001^b^
Late non-metastatic	171 (59.2)	98 (59.4)	
Late metastatic	79 (27.3)	64 (83.1)	
Type of comorbidity	N=343	N=200	
Chronic infectious disease (including HIV/AIDS)	16 (4.7)	12 (80)	0.0441^b^
Cardiovascular disease	74 (21.6)	36 (49.3)	
Gastrointestinal tract diseases	21 (6.1)	15 (71.4)	
Genitourinary diseases	2 (0.6)	1 (50)	
Renal disease	1 (0.3)	1 (100)	
Respiratory disease	1 (0.3)	0	
Metabolic disease	28 (8.2)	15 (55.6)	
Solid tumours (e.g. bone tumour)	4 (1.2)	3 (75)	
Number of comorbidities	N=343	N=200	
0	198 (57.7)	118 (61.5)	0.0040^b^
1	113 (32.9)	64 (57.7)	
2	28 (8.2)	16 (59.3)	
3	4 (1.2)	2 (50)	
hCCI	N=343	N=200	
0	198 (57.7)	118 (61.5)	0.0440^b^
1	102 (29.7)	55 (54.5)	
2–6	43 (12.5)	27 (65.9)	
a-hCCI	N=332	N=193	
0–1	90 (27.1)	56 (62.2)	0.0010^b^
2–4	203 (61.1)	110 (56.1)	
5–9	39 (11.8)	27 (69.2)	

a Percentages reported for mortality were calculated using the actual sample size for each variable as the denominator.

b Variables considered as potential covariates in the multivariable analysis.

In terms of the stage distribution of the disease at presentation, only a small proportion presented at an early stage (n=39 [13.5%]) compared with those who presented at a late stage, which was approximately five times more (n=171 [59.2%]) (Table 1). Of the subgroups, the median survival time was 9.02 y for the women in an early stage, 4.2 y for women with late non-metastatic stage presentation and <1 y for those who presented at a late metastatic stage.

### Cervical cancer mortality by comorbidity score

A total of 205 CC deaths were recorded during the study period, with a mean age at death of 59.8 y (SD 12.6). The overall CC mortality rate was 30.1 per 100 women-years, corresponding to a cumulative mortality of 60.3%. Cumulative mortality among women with no comorbidity was 61.5% (n=118), compared with 54.5% (n=55) among women with an hCCI of 1 and 65.9% (n=27) in women with an hCCI of 2–6 (Table 2).

**Table 2.** Unadjusted, age-adjusted and multivariable-adjusted HRs and CIs for CC mortality risk by hCCI.

Variables	CC mortality and mortality rate (per 100 women-years)^a^	hCCI						
		0 (n=118 [61.5%]) HR	1 (n=55 [54.5%])			2–6 (n=27 [65.0%])		
			Unadjusted HR (95% CI)	Model 1 (age HR (95% CI)	Model 2^b^ (aHR (95% CI)	Unadjusted HR (95% CI)	Model 1 (age HR (95% CI)	Model 2^b^ (aHR (95% CI)
All CC	205 (30.1)	1 (Ref.)	1.02 (0.74, 1.42)	0.95 (0.67 to 1.33)	1.13 (0.44, 2.93)	**1.68 (1.10 to 2.57)**	**1.57 (1.02** to **2.42)**	1.23 (0.55 to 2.73)
CC stage^c^								
Early-stage CC	39 (2.3)	1 (Ref.)	1.48 (0.47, 4.71)	1.13 (0.32 to 3.98)	1.39 (0.05 to 35.65)	3.39 (0.87 to 13.24)	3.46 (0.87 to 13.75)	11.02 (0.66 to 183.35)
Late non-metastatic stage CC	171 (14.3)	1 (Ref.)	1.39 (0.90 to 2.17)	1.42 (0.89 to 2.26)	0.29 (0.04 to 2.32)	1.00 (0.45 to 2.22)	1.01 (0.45 to 2.25)	0.18 (0.02 to 1.61)
Late metastatic stage CC	79 (9.2)	1 (Ref.)	0.57 (0.28 to 1.14)	0.51 (0.25 to 1.04)	1.48 (0.33 to 6.66)	**2.32 (1.23 to 4.39)**	**2.02 (1.04 to 3.91)**	**4.15 (1.69 to 10.18)**

a Crude total number of CC mortality cases recorded for each variable over the study period. The mortality rate, expressed per 100 women-years, was computed by dividing the total number of CC cases for each stage by the overall accrued time at risk for all study participants.

b Variables adjusted for include age, family history of CC in first-degree relatives, CC stage, anaemia and type of comorbidity.

c CC stage was removed from the adjusted model in the analysis by stage of the disease at diagnosis.

Statistically significant effect sizes are in bold.

Figure 1 provides a comprehensive graphical overview of mortality rates and mean survival times stratified by hCCI for both overall CC cases and across various CC stages. Notably, a distinctive upward trajectory in mortality rates emerged for an hCCI >1, with a notable increase from 29.1 per 100 women-years for hCCI 1 to 46.8 per 100 women-years for hCCI 2–6. This trend persisted across CC stages, reaching a peak of 70.3 per 100 women-years for late-metastatic CC with hCCI 2–6. In contrast, the corresponding mean survival times exhibited a marked decline with increasing hCCI >1 across all CC stages, reaching its nadir at 6 months for late metastatic CC when the hCCI was 2–6.

Relative to women with no comorbidity, mortality risk in women with hCCI 1 was unchanged in the unadjusted model and slightly reduced in the adjusted model, both of which were statistically insignificant. Among women with hCCI 2–6, there was approximately 70% and 60% increased risk of mortality in the unadjusted (HR 1.68 [95% CI 1.10 to 2.57]) and age-adjusted models (aHR 1.57 [95% CI 1.02 to 2.42]), respectively (Table 2).

When CC mortality was explored by stage of the disease at presentation, the mortality risk gradient by hCCI was most striking for late metastatic disease with hCCI 2–6, increasing more than twofold in the unadjusted model (HR 2.32 [95% CI 1.23 to 4.39]) compared with hCCI 0 to greater than fourfold in the adjusted model (aHR 4.15 [95% CI 1.69 to 10.18]) compared with hCCI 0 (Table 2).

When examining hCCI as a continuous predictor of CC mortality, a one-unit increase in hCCI was associated with an average 18% higher risk of mortality (age HR 1.18 [95% CI 1.06 to 1.33]). This risk became more pronounced, reaching 30%, particularly when diagnosed at the late metastatic stage (age HR 1.30 [95% CI 1.10 to 1.54]).

In the analysis investigating CC mortality by a-hCCI, the findings closely paralleled those obtained with hCCI, but with substantially larger effect sizes. A steep mortality risk gradient by a-hCCI was evident across all CC mortality (aHR 1.77 [95% CI 1.08 to 2.91] for a-hCCI 2–4 to 3.11 [95% CI 1.41 to 6.88] for a-hCCI 5–9). A similar pattern was observed for late metastatic disease, where the risk was more than twofold for a-hCCI 2–4 (aHR 2.44 [95% CI 1.17 to 5.10]) to almost sixfold for a-hCCI 5–9 (aHR 5.93 (95% CI 1.62 to 21.74) compared with a-hCCI 0–1 (Table 3).

**Table 3.** HRs and CIs for CC mortality risk by a-hCCI.

Variables	a-hCCI				
	0–1	2–4		5–9	
	HR (95% CI)	HR (95% CI)	aHR^a^ (95% CI)	HR (95% CI)	aHR^a^ (95% CI)
All CC	1 (Ref.)	1.08 (0.77 to 1.50)	**1.77 (1.08 to 2.91)**	**2.24 (1.41 to 3.58)**	**3.11 (1.41 to 6.88)**
CC stage^b^					
Early-stage CC	1 (Ref.)	0.74 (0.23 to 2.44)	1.31 (0.18 to 9.29)	2.29 (0.43 to 12.16)	5.13 (0.32 to 83.57)
Late non-metastatic stage CC	1 (Ref.)	0.95 (0.60 to 1.51)	1.15 (0.56 to 2.35)	1.67 (0.81 to 3.46)	1.86 (0.61 to 5.67)
Late-metastatic stage CC	1 (Ref.)	1.08 (0.59 to 1.98)	**2.44 (1.17 to 5.10)**	**3.15 (1.47 to 6.74)**	**5.93 (1.62 to 21.74)**

a Variables adjusted for include CC stage, family history of CC in first-degree relatives, anaemia and type of comorbidity.

b CC stage was removed from the adjusted model in the analysis by stage of the disease at diagnosis.

Statistically significant effect sizes are in bold.

## Discussion

Our study investigated the risk of mortality based on comorbidity status among CC patients in Lagos, Nigeria, revealing a clear risk gradient as comorbidity scores increase, particularly among women presenting with advanced-stage disease. Women with a higher hCCI (≥2) demonstrated significantly elevated mortality risks, both in crude and adjusted analyses, a pattern that persisted across all stages of the disease but was most pronounced in late metastatic presentations, where comorbidity exacerbated an already poor prognosis.

The identification of an elevated risk of CC mortality associated with increasing comorbidity scores aligns with the findings observed in various cancer types, as reported in previous studies.^6,9,19,20^ In our investigation, the distribution of CC mortality rates—27.8, 29.1 and 46.8 per 100 women-years—among women with an hCCI of 0, 1 and ≥2, respectively, revealed a progressive increase in CC mortality rates, irrespective of the disease stage. Moreover, when accounting for disease stage, these heightened rates were substantially increased, nearly doubling in magnitude.

While investigations into the impact of comorbidities on CC mortality risk remain sparse, existing studies were predominantly conducted in developed countries. For example, a recent risk stratification assessment involving 779 Medicare cancer patients in the USA identified CC, alongside factors such as increasing age, advanced metastatic stage and specific comorbidities (sepsis, renal failure, heart disease, hypertension and back pain), as strong predictors of unplanned acute care and hospitalization.^21^

Although our findings indicated there was no statistically significant alteration in mortality risk associated with an hCCI of 1 when compared with individuals without comorbidity (hCCI 0), the interpretation of this observation could be mixed. Previous research has reported conflicting results, with some studies^6^ revealing a higher incidence of late-stage cancer among patients with comorbidities, while others^6^ have identified a higher prevalence of comorbidity in patients diagnosed with early-stage diseases. Søgaard et al.^6^ argued in support of early cancer diagnosis among patients with comorbidities, attributing it to their increased interactions with the healthcare system. Other studies,^22,23^ such as those focusing on breast cancer, have highlighted the potential influence of the type of comorbid condition. However, our study reveals that the mortality rate and mean survival time, stratified by stage and comorbidity score, are notably similar for comorbidity scores of 1 and 0, particularly for late-stage cervical cancers.

In order to contextualize the impact of comorbidity within a clinical framework, we suggest the routine assessment and computation of comorbidity scores in addition to other baseline assessments for every CC patient. This nuanced approach is necessary for accurate stratification into prognostic categories, thereby influencing tailored treatment plans. Beyond the clinical realm, the incorporation of comorbidity assessments may serve as a valuable guide in offering effective psychosocial management of patients. By disclosing individualized risk profiles based on accompanying comorbidities, we anticipate an enhancement in patient counselling and engagement, fostering improved adherence to treatment, active participation in decision-making, collaborative efforts with the healthcare team and a potentially positive synergistic effect on overall treatment outcomes, both for CC and coexisting comorbidities.

While emphasizing the imperative of implementing additional measures to enhance clinical and supportive care for CC patients with any comorbidity, it is noteworthy that women with an hCCI >1 face an elevated risk of mortality, up to two to three times higher. This underscores the critical need for a strong multidisciplinary approach to treatment planning for this specific subgroup. The complexity introduced by comorbidity not only poses limitations on treatment options, but also significantly impacts the feasibility of curative interventions such as surgery, chemotherapy and radiotherapy, hindering the delivery of optimal care for cancer patients.^5,6,11^ This hesitancy towards aggressive treatment strategies is frequently attributed to concerns regarding increased treatment toxicity and postoperative or radiotherapeutic complications,^6^ as well as the potential for reduced treatment compliance.^6,19^

Although the prognostic influence of comorbidity scores on cancer outcomes has been demonstrated, there has been a noticeable paucity of efforts aimed at developing tailored management strategies. This prompted calls for the establishment of such protocols or the adaptation of existing ones, highlighting an area ripe for further clinical development and refinement.^11,24^ In addition, a noteworthy reduction in both overall all-cause and cancer-specific mortality risk was observed among cancer patients with other comorbidities when treated in specialized cancer centres compared with non-specialized tertiary care centers.^25^ Consequently, enhancing the availability and accessibility of more specialized cancer care centres where multidisciplinary care teams can more easily be formed in LMICs like Nigeria could offer significant potential for reducing mortality risks, particularly for late-stage multimorbid cancer patients.

Considering the high prevalence of comorbidities among cancer patients in Nigeria, as highlighted in our study and by other authors,^5,10^ it is imperative to address a few key public health considerations to improve mortality outcomes. The observed gradient in mortality risk with increasing comorbidity scores, especially among late-stage cases in our study, underscores the need for implementing robust population-based CC screening programs for early-stage cancer detection in Nigeria. This emphasis on early detection is especially important, as it has the potential to mitigate the added risk posed by concurrent comorbidities in these patients. The effectiveness of CC prevention, early detection and treatment strategies is well-established and has significantly reduced mortality rates by nearly 1% annually in many developed countries, especially the USa.^26^ These advancements form the cornerstone of the World Health Assembly's ambitious 90–70–90 strategy, aiming to achieve widespread impact by vaccinating 90% of eligible girls, screening 70% of eligible women by age 35 and ensuring prompt treatment for 90% of preinvasive and invasive CC cases by the year 2030.^27^

### Study strengths and limitations

This study used clinical data characterized by high reliability and accuracy, a result of meticulous cross-validation of patient information through repeated reviews conducted by multiple healthcare professionals during the course of clinical management. The examination of comorbidity scores in various formats, such as hCCI and a-hCCI, consistently yielded corroborative findings, enhancing the overall reliability of the study's conclusions. Furthermore, the incorporation of histological reports, assessed by pathologists to confirm CC diagnoses, not only bolstered diagnostic accuracy, but also contributed to the precise computation of follow-up times. Given the limited research in the context of developing regions, our study bridged the knowledge gap by presenting mortality risk levels associated with the comorbidity burden of CC patients in Nigeria. By offering insights into individualized risk stratification, our findings contribute to a broader understanding of CC outcomes in resource-constrained settings.

While our study has provided valuable insights, it is important to acknowledge several limitations that may impact the interpretation and application of its findings. The absence of a population-based death register posed a challenge to confirm the cause of death within the study cohort. The availability of such a database would have enabled a more nuanced analysis, allowing us to explore the influence of comorbidity on both all-cause and CC-specific mortality. Another limitation was the inability to accurately determine and access all records of CC patients during the study period, reflecting the need for improved medical record-keeping and data management systems to enhance the quality and reproducibility of future research in resource-limited settings. Additionally, the institutional base of our data introduces a degree of homogeneity, potentially limiting the generalizability of our results to broader populations. Given the observational nature of our study, we cannot attribute causality to the relationship between comorbidity and CC mortality.

## Conclusions

Our investigation into the role of comorbidity as a predictor for mortality risk stratification among CC patients in Lagos, Nigeria, reveals a notable association between an increasing comorbidity score and CC stage at diagnosis and an increased risk of mortality. The incorporation of comorbidity scoring as a routine component in the initial comprehensive clinical assessment of every CC patient emerges as a crucial aspect, holding the potential to impact prognostic categorization, treatment strategies and patients’ psychosocial management.

We recommend that a multidisciplinary team be involved in the management of CC patients with comorbidities in hospitals in Nigeria. In addition to primary oncology care, the multidisciplinary team could include cardiologists, endocrinologists and other relevant specialist practitioners to comprehensively address the complex needs of these patients. The integration of specialists ensures that both the cancer and coexisting conditions are managed concurrently, improving CC patients’ outcomes.

Future research endeavours are essential to validate these findings in diverse cohorts, evaluate the feasibility of integrating comorbidity metrics into clinical cancer care and assess their implications for patient outcomes and health system efficiency in Nigeria.

## Data Availability

The data underlying this article will be shared upon reasonable request to the corresponding author.
